# Effects of Resveratrol Supplementation on Bone Growth in Young Rats and Microarchitecture and Remodeling in Ageing Rats

**DOI:** 10.3390/nu6125871

**Published:** 2014-12-16

**Authors:** Alice M. C. Lee, Tetyana Shandala, Long Nguyen, Beverly S. Muhlhausler, Ke-Ming Chen, Peter R. Howe, Cory J. Xian

**Affiliations:** 1 Sansom Institute for Health Research, School of Pharmacy and Medical Sciences, University of South Australia, Adelaide, SA 5001, Australia; E-Mails: alice.lee@unisa.edu.au (A.M.C.L.); tetyanas@geneworks.com.au (T.S.); long.nguyen@unisa.edu.au (L.N.); Beverly.Muhlhausler@adelaide.edu.au (B.S.M.); 2 FOODplus Research Centre, School of Agriculture Food and Wine, Waite Main Building, University of Adelaide, SA 5064, Australia; 3 Institute of Orthopaedics, Lanzhou General Hospital, Lanzhou Command of People’s Liberation Army, Lanzhou 730050, China; E-Mail: chenkm@lut.cn; 4 Clinical Nutrition Research Centre, University of Newcastle, Callaghan, NSW 2308, Australia; E-Mail: peter.howe@newcastle.edu.au

**Keywords:** resveratrol, bone volume, osteoporosis, peak bone mass

## Abstract

Osteoporosis is a highly prevalent skeletal disorder in the elderly that causes serious bone fractures. Peak bone mass achieved at adolescence has been shown to predict bone mass and osteoporosis related risk fracture later in life. Resveratrol, a natural polyphenol compound, may have the potential to promote bone formation and reduce bone resorption. However, it is unclear whether it can aid bone growth and bone mass accumulation during rapid growth and modulate bone metabolism during ageing. Using rat models, the current study investigated the potential effects of resveratrol supplementation during the rapid postnatal growth period and in late adulthood (early ageing) on bone microarchitecture and metabolism. In the growth trial, 4-week-old male hooded Wistar rats on a normal chow diet were given resveratrol (2.5 mg/kg/day) or vehicle control for 5 weeks. In the ageing trial, 6-month-old male hooded Wistar rats were treated with resveratrol (20 mg/kg/day) or vehicle for 3 months. Treatment effects in the tibia were examined by μ-computer tomography (μ-CT) analysis, bone histomorphometric measurements and reverse transcription-polymerase chain reaction (RT-PCR) gene expression analysis. Resveratrol treatment did not affect trabecular bone volume and bone remodeling indices in the youth animal model. Resveratrol supplementation in the early ageing rats tended to decrease trabecular bone volume, Sirt1 gene expression and increased expression of adipogenesis-related genes in bone, all of which were statistically insignificant. However, it decreased osteocalcin expression (*p* = 0.03). Furthermore, serum levels of bone resorption marker C-terminal telopeptides type I collagen (CTX-1) were significantly elevated in the resveratrol supplementation group (*p* = 0.02) with no changes observed in serum levels of bone formation marker alkaline phosphatase (ALP). These results in rat models suggest that resveratrol supplementation does not significantly affect bone volume during the rapid growth phase but may potentially have negative effects on male skeleton during early ageing.

## 1. Introduction

Osteoporosis is a disease that is characterized by a progressive decrease in bone mass and subsequently an increase in fracture risk [1]. Osteoporotic fractures are a major cause of morbidity and disability in the elderly [2]. Despite recent advances in treatments for osteoporosis [3,4], due to their side effects, risks and cost concerns, more studies are needed to investigate potential roles for natural substances or nutraceuticals in preventing or delaying the onset of osteoporosis.

The importance of peak bone mass (accrued by late adolescence or young adulthood) in relation to fracture risk has been studied extensively, and developing strategies to enhance peak bone mass has been suggested to be an important means of reducing the risk of osteoporosis in later life. It has been predicted that a 10% increase in peak bone mass would delay the onset of osteoporosis by 13 years [5]. Furthermore, epidemiological studies have shown that a 10% increase in peak bone mass could also be predicted to reduce the risk of fracture by 50% in women after menopause [5,6,7]. Since bone loss continues with ageing, the bone mass of individuals later in life largely depends upon the original peak bone mass achieved during the skeletal growth phase and the subsequent rate of bone loss. Thus, preventative means against osteoporosis should be aimed at increasing the peak bone mass during growth and reducing the rate of bone loss during ageing.

While bone mass is known to be largely controlled by genetics, it is also influenced by life-style factors such as physical loading and nutrient intake [8]. A number of nutritional supplements, including vitamins, calcium, protein, and some individual amino acids, have been shown to promote bone formation and/or inhibit osteoporosis [9,10]. In recent years, the potential benefits to bone health of a natural compound called resveratrol have attracted attention. Resveratrol is a polyphenolic phytoestrogen that naturally occurs in the skin of red grapes, various other fruits, peanuts and root extracts of the weed *Polygonum cuspidatum* [11,12]. Resveratrol has been identified as a potent activator of Sirtuin 1 (Sirt1), which is also known as nicotinamide adenine dinucleotide (NAD)-dependent deacetylase [13], and dietary supplementation with this compound can mimic the benefits of caloric restriction in mice fed a high fat diet [13]. It has also been shown that resveratrol exhibits anti-oxidant, anti-inflammatory, and anti-catabolic properties [14,15]. Related to its effects on bone health, *in vitro* studies have shown that resveratrol may promote formation and activity of osteoblasts (bone-forming cells) and antagonize differentiation and function of osteoclasts (bone-resorbing cells) [16,17,18]. *In vitro* studies have shown that resveratrol dose dependently increases alkaline phosphatase (ALP) activity (a biomarker for osteoblast differentiation), indicating its ability to stimulate differentiation of osteoblasts [19]. Furthermore, activation of Sirt1 has been shown to downregulate *in vitro* preadipocyte proliferation and adipogenic differentiation by inhibiting the transcription activity of adipogenesis transcription factors peroxisome proliferator-activated receptor gamma (PPARγ) and CCAAT/enhancer-binding protein alpha (C/EBPα) [20,21,22,23]; and concomitant inhibition of PPARγ increases the expression of osteoblastic markers including runt-related transcription factor 2 (Runx2), osteocalcin (OCN), alkaline phosphatase (ALP), and osteopontin [16,24,25].

Despite the above *in vitro* studies on potential osteotrophic effects of resveratrol, its potential function *in vivo* in regulating bone formation and remodeling is less clear. Previously, resveratrol supplementation was shown to be protective against disuse-induced bone loss in hindlimb suspension in young [26] and mature male rats, and resveratrol treatment appeared to prevent the decline in bone microarchitecture in aged rats [27]. Resveratrol was also reported to increase epiphyseal bone mineral density and inhibit the decrease of femur calcium content in ovariectomized rats [28]; and its phytoestrogenic effect in ovariectomized rats was equivalent to the effects of hormone replacement therapy, further suggesting its potential bone health benefits during estrogen deficiency [29,30]. However, it is unclear whether resveratrol supplementation can help with bone growth and bone mass accumulation during rapid growth in early life and modulate bone metabolism during early ageing, both of which are likely to be important for reducing the risk of osteoporosis and associated structures. Using rat models, the current study addresses whether resveratrol supplementation can help with bone mass accumulation during rapid growth in early life and prevent bone loss by modulating bone metabolism during early ageing.

## 2. Experimental Section

### 2.1. Animal Trials and Specimen Collection

All procedures were approved by the Animal Ethics Committees of the University of South Australia and Institute of Medical and Veterinary Sciences (IMVS, Adelaide, South Australia). Male Hooded Wistar rats (IMVS, Adelaide, South Australia) at 4 weeks of age for the youth study were on a normal rat chow diet (Laucke Mills, Daveyton, Australia) and were divided into groups receiving vehicle control or resveratrol supplementation. ResVida^®^, the purest form of *trans*-resveratrol (DSM, Basel, Switzerland), was fully dissolved in 100% ethanol and spotted onto small pieces of dried apple chips to allow easy consumption for the rats. After ethanol evaporation, loaded apple chips were given to and orally consumed by rats (one piece per rat per day), with the loaded resveratrol at a specific dose based on body weight. Vehicle control rats also received small pieces of apple chips spotted with ethanol only. Our prior pilot study had shown that resveratrol given to fast growing rats at 10 mg/kg/day for 14 days significantly shortened growth plate height suggesting potential toxicity at this dosage in fast growing rats (data not shown). Therefore, in the current study, resveratrol was given at 2.5 mg/kg/day (which has been previously shown to have cardioprotective effects [31,32]) for 5 weeks (*n* = eight rats, for both resveratrol and control groups).

For the early ageing trial, male Hooded Wistar rats at 6 months of age were on a normal chow diet before resveratrol supplementation. Resveratrol loaded apple chips were prepared in the same way as mentioned above. Resveratrol was given for 3 months at a dose of 20 mg/kg/day (*n* = seven rats, for both resveratrol and control groups). This dosage has been shown to be non-toxic to adult rats given for 4 weeks [33].

At the end of each trial, following euthanasia by CO_2_ overdose, the left tibia was collected, fixed in 10% formalin for 24 h, and used for micro-computed topographical μ-computer tomography (μ-CT) analyses (see below). After μ-CT scanning, bones were then decalcified in Immunocal (Decal Corp, Tallman, NY, USA) at 4 °C, bisected, processed, and embedded in paraffin wax for histological studies. Growth plate and metaphyseal region were collected from the right tibia and stored at −80 °C until RNA isolation.

### 2.2. Bone Volume and Micro-Architecture

To examine treatment effects on bone volume and structure of long bones, the left proximal tibia bone micro-architecture of both young and early ageing rats were analyzed using a high-resolution μ-CT system (Skyscan 1172, Antwerp, Belgium) which produces multiple X-ray transmission images. Cross sectional images of the object were then reconstructed by a modified Feldkamp cone-beam algorithm, creating a complete 3-D representation of internal microstructure and density. The analysis was limited to a 2 mm region of interest of trabecular network starting from 2 mm below the growth plate. Cortical bone volume was limited to a region of interest of 2 mm below the secondary spongiosa containing only cortical bone. The resolution of scanning was 11.2 μm/pixel, which provides sufficiently detailed information to calculate trabecular bone volume (Bone volume/Tissue volume or BV/TV, %) and cortical bone volume (mm^3^).

### 2.3. Histomorphometric Analysis of Metaphysis

Paraffin longitudinal sections of 5-μm thickness (four serial sections per rat, 100 μm apart) mounted on SuperFrost Plus glass slides were dewaxed and stained with a routine haematoxylin and eosin (H & E) staining procedure. Stained sections were used for histomorphometric measurements of growth plate thickness, primary spongiosa heights, in both young and early ageing rats; densities of osteoblasts and bone marrow adipocytes were measured in early ageing rats only as described [34,35]. Primary spongiosa heights were obtained by measuring the heights between the end of growth plate and the top of secondary spongiosa. On H & E-stained tibial sections, osteoblasts on the trabecular surface in the secondary spongiosa of the metaphysis were counted and expressed as number of cells/unit length trabecular bone perimeter. To identify osteoclasts, sections were stained for tartrate-resistant acidic phosphatase (TRAP, a marker for osteoclasts) using established methods and reagents (Sigma, Sydney, NSW, Australia). TRAP^+^ cells were counted by light microscopy and expressed as number of TRAP^+^ cells/unit length trabecular perimeter in the secondary spongiosa. Adipocytes within bone marrow area were counted in four random images within the lower secondary spongiosa region, and expressed as adipocyte number per mm^2^ marrow area.

### 2.4. Quantitative RT-PCR (qRT-PCR) Analysis of Gene Expression

Real time quantitative reverse transcription-polymerase chain reaction (RT-PCR) assay was used to examine treatment effects on mRNA expression of Sirt1 and osteogenesis and adipogenesis regulatory genes in early ageing rats. To extract RNA, frozen metaphyseal bone specimens were ground using a mortar and pestle and liquid nitrogen, and total RNA was extracted with TRI reagent (Sigma-Aldrich, New South Wales, Australia). To generate the template for PCR amplification, 2 μg of metaphyseal RNA was reverse transcribed into cDNA using the high capacity RNA-to-cDNA kit (Applied Biosystems, Foster City, CA, USA). This cDNA was used to determine the mRNA expression for the genes of interest by quantitative real-time PCR as previously described using gene specific primers detailed in Table 1 designed using Primer Express Software version 2.0 (Applied Biosystems, Victoria, Australia) and cyclophilin-A (CycA) as the housekeeping control loading gene. SYBR green PCR assays for each target molecule and internal reference CycA were performed in duplicate on these cDNA samples in a 10 μL reaction using Applied Biosystems 7500 FAST 96-well PCR machine. From the amplification curves, relative expression was calculated using the comparative *C*_t_ (2^−Δ*C*t^) method, with CycA serving as the endogenous control and the expression data as a ratio (target gene/CycA).

**Table 1:** Forward and reverse primer sequences used in the polymerase chain reaction (PCR) study.

Gene	Forward Primer (5′–3′)	Reverse Primer (5′–3′)
CycA	GAGCTGTTTGCAGACAAAGTTC	CCTGGCACATGAATCCTGG
Osterix	GCTTTTCTGTGGCAAGAGGTTC	CTGATGTTTCTCAAGTGGTCG
Osteocalcin	AAGCCTTCATGTCCAAGCAGG	AGGCGGTGTTGAAGCCATACT
C/EBPα	TCGCCATGCCGGGAGAACTCTAAC	CTGGAGGTGGCTGCTCATCGGGG
Sirt 1	AGA AACAATTCCTCCACCTGA	GCTTTGGTGGTTCTGAAAGG
FABP4	GGAATTCGATGAAATCACCCC	TGGTCGACTTTCCATCCCACT

CycA, cyclophilin-A; C/EBPα, CCAAT/enhancer-binding protein alpha; Sirt 1, Sirtuin 1; FABP4, fatty acid-binding protein 4.

### 2.5. Measurement of Collagen Type I Cross-Linked C-Telopeptide (CTX-1) and Alkaline Phosphotase (ALP) in Serum

Enzyme-linked immunosorbent assay (ELISA) was performed to measure the levels of bone resorption marker CTX-1 in serum of control *vs.* treated rats using ELISA kit for CTX-1 (Cusabio Life Science, Hubei, China). Serum levels for bone formation marker ALP were measured using Konelab 20XT clinical chemistry analyzer (Thermo Scientific, Vantaa, Finland).

### 2.6. Statistical Analysis

Data are presented as mean ± standard error of mean (SEM). Data from μ-CT, histological analyses, and RT-PCR were evaluated by Student’s unpaired *t*-test with Tukey’s *post hoc* tests. *p* < 0.05 was considered statistically significant in all analyses. Analyses were performed using Prism version 6 (Graphpad Software Inc., La Jolla, CA, USA).

## 3. Results

### 3.1. Effects of Resveratrol Supplementation in Young Animals

**Figure 1:**
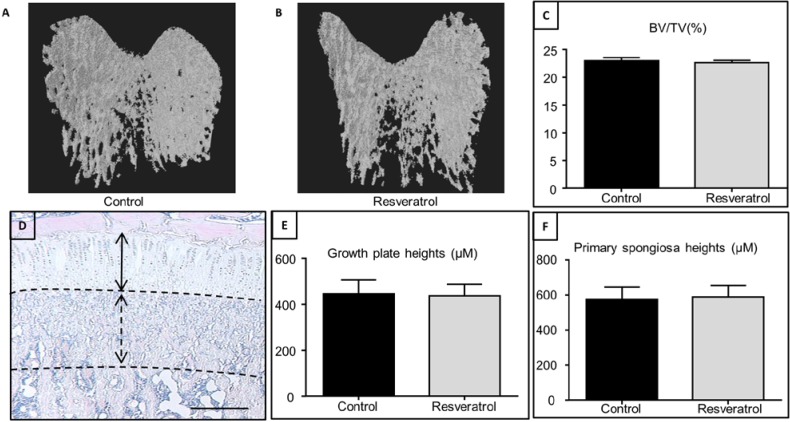
The effects of resveratrol supplementation on growth plate, primary spongiosa heights, and bone volume in the tibia of young rats. 3D reconstruction images showing trabecular bone in (**A**) a control rat and (**B**) a resveratrol treated rat; (**C**) The effects of resveratrol supplementation on trabecular bone volume/tissue volume (BV/TV) from μ-CT analysis in young animals; (**D**) Histological analysis showing H & E stained section showing the growth plate and primary spongiosa of a control rat (bar = 500 μm); (**E**) Measurements of growth plate thickness; (**F**) Measurements of primary spongiosa heights.

Effects of resveratrol supplementation at 2.5 mg/kg from 4 to 9 weeks of age on bone structure and volume were examined by μ-CT and histomorphometry. μ-CT analysis revealed no significant differences in trabecular bone structures and volume (BV/TV, %) between the resveratrol and control groups (Figure 1A–C). As assessed by histological analysis, there was no effect of resveratrol supplementation on growth plate thickness (Figure 1D,E) and primary spongiosa heights (Figure 1F).

### 3.2. Effects of Resveratrol Supplementation on Bone Structure and Osteogenesis Regulatory Genes in Ageing Rats

**Figure 2:**
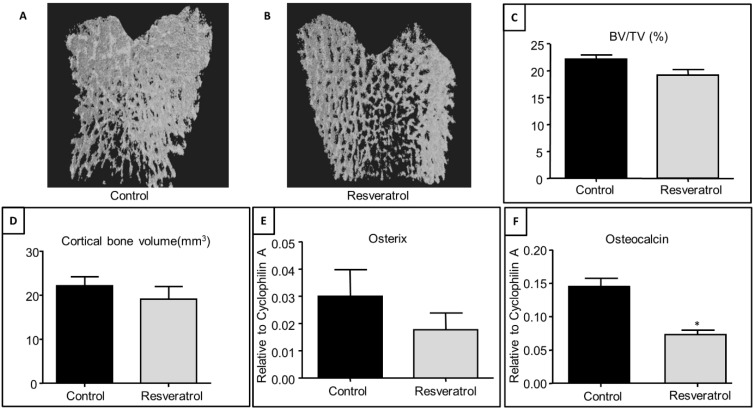
Effects of resveratrol supplementation on the trabecular bone structure and levels of mRNA expression of osteogenesis-related factors in the metaphysis of the tibial bone of ageing rats. 3D reconstruction images showing trabecular bone in (**A**) a control rat and (**B**) a resveratrol treated rat; (**C**) Effects on trabecular BV/TV from μ-CT analysis; (**D**) Effects on cortical bone volume from μ-CT analysis; (**E**) RT-PCR gene expression analyses for osterix (*p* = 0.34); (**F**) RT-PCR gene expression analyses for osteocalcin (*p* = 0.02). * *p* < 0.05 compared to control group, *n* = 7.

Effects on bone structure and volume of resveratrol supplementation (for 3 months at 20 mg/kg body weight) were also investigated in the ageing rats (at 9 months of age). μ-CT analysis revealed no significant differences in the trabecular bone structures (Figure 2A,B) and volume (BV/TV, %) in the metaphysis between the treatment groups, although resveratrol treatment caused an 8% reduction in the trabecular bone volume (*p* = 0.09) (Figure 2C). There was no significant effect of resveratrol treatment on cortical bone volume (Figure 2D).

At the gene expression level, male rats treated with resveratrol between 6 and 9 months of age tended to show in a reduced mRNA expression of the osteogenic transcription factor, osterix, compared to control (vehicle-treated) animals (Figure 2E) (*p* = 0.32). Resveratrol treatment from 6 to 9 months of age also exhibited a 50% reduction in mRNA expression of the bone-matrix protein, osteocalcin (*p* < 0.05, Figure 2F) at the end of the intervention.

### 3.3. Effects on Osteoclast, Osteoblast, and Adipocyte Densities in the Secondary Spongiosa of Ageing Rats

**Figure 3:**
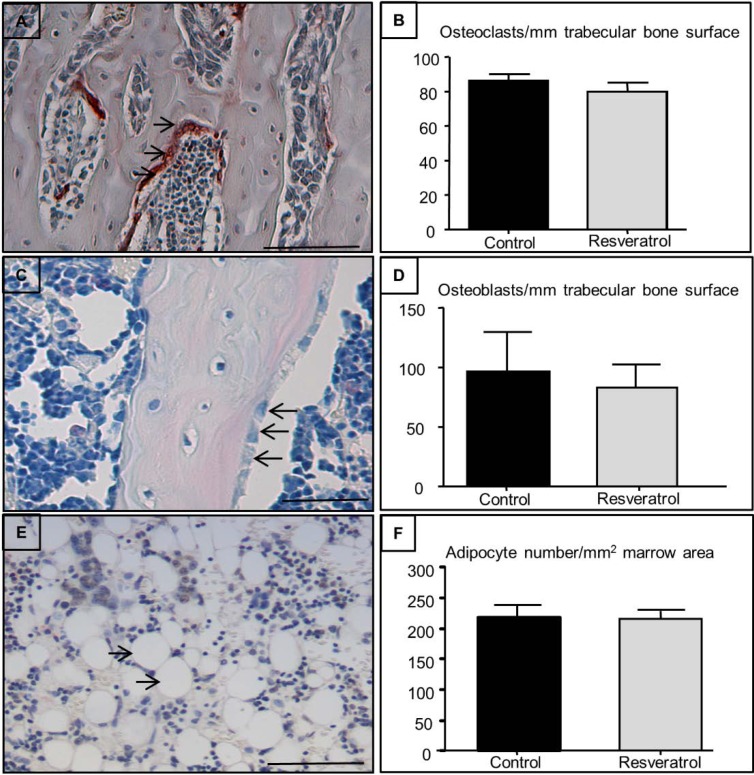
Effects of resveratrol supplementation on osteoclast, osteoblast, and adipocyte densities within the secondary spongiosa in early ageing rats. Histological analyses demonstrating (**A**) TRAP stained section of a control tibial bone showing stained osteoclasts (cells stained red containing three + nuclei as indicated by arrows) (bar = 100 μm); (**B**) Osteoclast density at the secondary spongiosa (*n* = 7); (**C**) H & E-stained section showing osteoblasts in secondary spongiosa as indicated by arrows (bar = 125μm); (**D**) Osteoblast density within the secondary spongiosa (*n* = 7); (**E**) H & E-stained section showing adipocytes in lower secondary spongiosa as indicated by arrows (bar = 100 μm); (**F**) Adipocyte density within the lower secondary spongiosa (*n* = 7).

Effects of resveratrol treatment on osteoclast (Figure 3A,B), osteoblast (Figure 3C,D), and adipocyte (Figure 3E,F) densities in secondary spongiosa in ageing rats were also measured. There were no significant differences in osteoclast and osteoblast densities in the secondary spongiosa between control and resveratrol-treated rats (Figure 3B,D). Resveratrol treatment also had no significant effects on adipocyte density when compared with the control group (Figure 3F).

### 3.4. Expression of Adipogenesis Regulatory Genes and Sirt 1 in Early Ageing Rats

**Figure 4:**
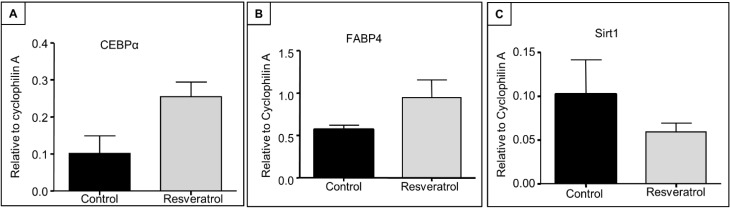
Effects of resveratrol supplementation on mRNA expression levels of adipogenesis-related factors and Sirtuin 1 (Sirt1) in early ageing rats. RT-PCR gene expression analyses at tibial metaphysis for (**A**) C/EBPα (*p* = 0.06); (**B**) FABP4 (*p* = 0.34) and (**C**) Sirt1 (*p* = 0.36) (*n* = 7). C/EBPα, CCAAT/enhancer-binding protein alpha; FABP4, fatty acid-binding protein 4.

qRT-PCR analysis of adipogenesis regulatory genes revealed that, although not statistically significant, the mRNA expression of C/EBPα tended (*p* = 0.06) to increase in the bone after resveratrol treatment (Figure 4A). A similar trend was also seen (towards an increase) in the expression of FABP4 (*p* = 0.34, Figure 4B). However, qRT-PCR analysis did not show any significant changes in mRNA expression of Sirt1 with resveratrol supplementation when compared to the control group (*p* = 0.36, Figure 4C).

### 3.5. Effects of Resveratrol Supplementation in Serum Levels of CTX-1 and ALP in Ageing Rats

**Figure 5:**
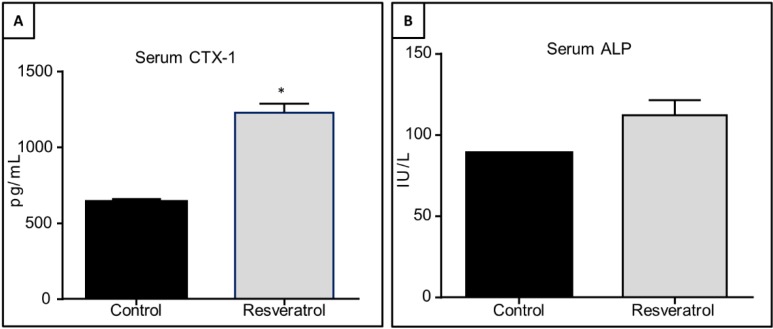
Effects of resveratrol supplementation on serum levels of bone resorption marker collagen type I cross-linked C-telopeptide (CTX-1) and bone formation marker alkaline 12hosphatase (ALP) in the ageing rats. Serum levels of (**A**) CTX-1 (*p* = 0.02); (**B**) ALP (*p* = 0.36).

The levels of bone resorption (CTX-1) and formation (ALP) markers in ageing rats in response to resveratrol treatment were examined. Serum CTX-1 levels were significantly upregulated in resveratrol treated animals (*p* = 0.02, Figure 5A). However, there were no significant changes in the serum levels of ALP (*p* = 0.36, Figure 5B).

## 4. Discussion

The deterioration of the skeleton with advancing age affects an increasingly larger population and contributes significantly to the associated epidemic of nontraumatic fractures. Achieving maximum bone density and minimizing bone loss have been recommended as major strategies to prevent or delay the occurrence of osteoporosis [36]. Although resveratrol has been suggested to possess a wide range of health benefits including its potential bone protective effects, no previous studies have determined whether supplementation with this natural compound could optimize attainment of bone mass during growth and/or reduce bone loss in early ageing (at 6–9 months). In the current study, we have demonstrated that oral resveratrol supplementation for 5 weeks during the rapid growth period has no effects on the long bone structure and volume in young rats. When given for 3 months in the early ageing period (6–9 months of age), resveratrol supplementation may have negative effects on the ageing male skeleton as it tends to reduce trabecular bone volume and gene expression of bone formation marker osteocalcin in the bone whilst increasing the serum level of the bone resorption marker CTX-1.

### 4.1. Effects of Resveratrol Supplementation on Peak Bone Mass

While *in vitro* studies have shown that resveratrol can dose-dependently increase proliferation and differentiation of osteoblastic MC3T3-E1 cells [17] and can promote human mesenchymal stem cell commitment to the osteogenic lineage through the master osteogenic transcription factor RUNX2 [24,37], our current study demonstrated that resveratrol supplementation for 5 weeks during the rapid growth period in male rats had no significant effects on growth plate thickness, primary spongiosa heights and trabecular bone volume by the end of treatment, suggesting a lack of effect of resveratrol supplementation on the bone mass outcome in growing rats. These findings are in agreement with a previous study using an isoflavone-enriched diet containing soybean extract, daidzein, genistein, and equol in 6-week-old growing female pigs, which also found no significant changes in the growth plate, mineralization or osteoblast/osteoclast densities in long bones [38]. Furthermore, another study showed no differences in cortical bone measurements in growing rats given different doses of resveratrol (1, 4, 10, 40, and 100 μg/day) [39]. On the other hand, positive phytoestrogen effects of isoflavone on reducing ovariectomy-induced bone loss have been observed in female rats, which may suggest potential physiological differences (between normal growth and estrogen deficiency) in responding to isoflavone or resveratrol treatments [40,41]. Further studies to evaluate potential effects of resveratrol supplementation on the growing skeleton in different doses or treatment periods are warranted.

### 4.2. Potential Effects of Resveratrol on Ageing-Induced Bone Loss

While the anti-resorptive bisphosphonates have now become a major approach to treat osteoporosis, there has been an increased interest among researchers and the public to use osteotrophic natural substances or nutraceuticals to prevent bone loss or osteoporosis [42,43]. Our study has demonstrated that both trabecular and cortical bone structure were not significantly altered by 3 months of resveratrol supplementation in male rodents between 6 and 9 months of age despite a trend of reduction in trabecular bone volume. Furthermore, although no changes in the densities of osteoblasts and osteoclasts on trabecular bone surfaces were detected, serum CTX-1 levels were significantly elevated in animals supplemented with resveratrol despite maintenance of serum ALP levels. The increase in this bone turnover marker suggests that resveratrol supplementation may potentially have a negative effect on the bone. In contrast, in previous studies in ovariectomized adult rats, oral supplementation with resveratrol at 45 mg/kg a day for 90 days was shown to be able to reduce bone turnover and reverse bone loss due to its phytoestrogen properties [44]. Another previous study in ovariectomized rats showed that treating animals daily with 0.7 mg/kg resveratrol by oral gavage could increase epiphysis bone mineral density and inhibit ovariectomy-induced reduction in bone calcium content in the femur [28]. Additionally, a study which utilized male mice at 12 months of age demonstrated that resveratrol supplementation for 18 months significantly improved cortical tissue mineral density in the distal femur with a trend of increase in bone strength [28]. These previous studies and our current work suggest that bone protection outcomes of resveratrol may depend on the bone loss models involved. In support of this, previous studies have demonstrated that ageing-related bone loss in male long bones may be more pronounced at the age of 24–27 months [45,46,47]. This can be further supported by another study which demonstrated significantly improved femoral bone volume as well as bone quality in 22-month-old rats given resveratrol for 10 weeks [48].

While resveratrol may reduce bone loss in ovariectomized and aged rats as discussed above, it appears to have no protective effects and even potentially negative effects on bone health during early ageing (6–9 months of age). Interestingly, a recent study showed that male rats aged 33 months given a daily oral gavage of 12.5 mg/kg body weight of resveratrol did not have significant effects on bone mineral density, bone mineral content, bone mineral area and strength [49]. Additionally, Sehmisch *et al.* reported that mature ovariectomized rats provided diets supplemented with 5 or 50 mg/kg body weight of resveratrol for 12 weeks had no effect on tibial total bone mineral density, cortical or trabecular bone and bone strength [49]. Consistently, a hindlimb suspension study using 6-month-old rats with resveratrol given at 12.5 mg/kg/day for 21 days showed no bone protective effects and did not attenuate trabecular bone deterioration in hindlimb suspended rats. Furthermore, suggesting a possible detrimental effect of resveratrol supplementation to the bone in this model, hindlimb suspended rats with resveratrol treatment showed significantly lower tibial bone mineral content, calcium content, and cortical thickness when compared to rats without supplementation [50]. These results, in accordance with our data, suggest that there is a need to understand the bioavailability and pharmacokinetics of resveratrol.

Up to 90% of resveratrol is reported to be absorbed rapidly following oral administration [45,46,47,51]. Resveratrol is first evident in plasma 15 min after oral administration and peaks after 30 min [52,53]; it is quickly metabolized in the intestine and liver into glucuronide and sulfate conjugates [54]. Resveratrol and its metabolites are widely distributed in many organs, particularly the gastrointestinal intestinal tract, liver, kidney, heart, and lungs [55,56]. Furthermore, *in vivo* evidence indicates that resveratrol has low bioavailability due to rapid metabolism in the body [56]. Various other studies have investigated clinical doses of resveratrol and have demonstrated that the effective human dose for resveratrol ranges from 10 to 120 mg/day [57,58]. Due to the discrepancies between *in vitro* and *in vivo* studies and confusion due to differences in models, dosage, and species, it is difficult to extrapolate the insights of previous experiments to our current study. Therefore, further studies are required to identify any potential beneficial effects of resveratrol supplementation in maximizing bone mass and preventing osteoporosis.

### 4.3. Effects of Resveratrol Supplementation on Gene Expression in the Bone

It has been well established that adipocytes and osteoblasts are derived from common progenitor cells, the mesenchymal stem cells, in the bone marrow [59,60]. It has been shown that resveratrol can reduce the synthesis of lipids in 3T3-L1 adipocytes [20] and pig primary adipocytes [61]. Several *in vitro* studies have proposed that the reduced adipogenic potential following resveratrol supplementation is due to the activation of Sirt1 [20,62]. These studies indicate that resveratrol supplementation dose-dependently down regulates PPARγ, C/EBPα, and other genes mediating adipogenesis and fat storage through Sirt1 activation. Contrary to these *in vitro* studies, the present *in vivo* study showed no significant effects of resveratrol treatment on Sirt 1 gene expression in early ageing rats, and even a trend towards a down-regulation. Interestingly, following resveratrol treatment, there was a trend of up regulation in the expression of adipogenesis markers (C/EBPα and FABP4), but a significant reduction in mRNA expression of the bone formation marker osteocalcin in the tibia of early ageing male rats. The apparent reciprocal relationship between the bone formation marker (osteocalcin) and adipogenesis markers (C/EBPα and FABP4) may suggest that, in this animal model of early ageing, resveratrol supplementation given at the dose and duration used in this study may not be beneficial to the bone, and it may even suppress bone formation or increase bone resorption. Further studies are required to elucidate the effect and action mechanisms of resveratrol in the bone.

## 5. Conclusions

In conclusion, even though the available evidence of resveratrol on bone health remains too sparse to be conclusive, we have demonstrated that resveratrol supplementation in our rapidly growing rats causes no obvious changes in bone structure and volume. However, our early ageing rat model demonstrated potential negative effects of resveratrol supplementation showing a trend but non-significant reduction in trabecular bone volume, which was accompanied by a reduction in the expression of bone formation marker, osteocalcin, and a trend towards increased expression of adipogenesis-related genes in the metaphysis. Furthermore, this was accompanied by elevated levels of serum resorption marker CTX-1 despite the lack of changes in osteoclast density on the trabecular bone surface. As the current study was limited to examining the effects of resveratrol treatment at a single time point, we cannot exclude that different effects would emerge had we continued the supplementation for a longer period or with a different method of delivery, and future studies should be carried out with an ageing model at various time points, different doses, and at older ages to monitor the extent of bone loss and changes in bone metabolism.
